# Vascular Endothelial Function, Carotid Intima–Media Thickness and Coronary Artery Calcification in Women

**DOI:** 10.3390/jcm15114087

**Published:** 2026-05-25

**Authors:** Maciej Koźlik, Marta Chamera, Jonasz Osiecki, Anna Kaźmierska, Jakub Kufel, Iga Paszkiewicz, Marta Bujak, Szymon Ładziński, Maciej Kaźmierski

**Affiliations:** 1 Department of Cardiology and Structural Heart Diseases, Medical University of Silesia, 40-635 Katowice, Poland; maciej.kozlik5@gmail.com (M.K.); osiecki.jo@gmail.com (J.O.); igapaszkiewicz.ip@gmail.com (I.P.); martagoral5@wp.pl (M.B.); szymon.ladzinski@gmail.com (S.Ł.); kazmierski.maciej@gmail.com (M.K.); 2 Department of Radiodiagnostics, Invasive Radiology and Nuclear Medicine, Faculty of Medical Sciences in Katowice, Medical University of Silesia, 40-752 Katowice, Poland; jakubkufel92@gmail.com

**Keywords:** carotid intima–media thickness, coronary disease, risk factors, tomography, vascular endothelium, women

## Abstract

**Background**: Atherosclerosis is a degenerative-proliferative disease that leads to lesions primarily in the tunica intima and media of the arteries. Atherosclerotic plaques undergo progressive calcification, and the hydroxyapatite deposited within them absorbs X-rays. The coronary artery calcification score (CAC-score) can be assessed using computed tomography. Intima–media thickness (IMT) and endothelial function, evaluated by flow-mediated dilatation (FMD) of the brachial artery, can be measured using ultrasound. This study aimed to assess the relationship between CAC-score, atherosclerosis risk factors, IMT, and FMD in women, with particular emphasis on the comparison of IMT measurement sites. **Methods**: The study included 124 women divided into three groups based on CAC-score. The following parameters were evaluated: risk factors for coronary artery disease (CAD), FMD, and IMT. CAD risk factors included age, BMI, smoking status, hypertension, diabetes, and lipid disorders, which were obtained from medical history. **Results**: A significant positive correlation was observed between CAC-score and IMT of the common carotid artery in women. IMT measured at the carotid bifurcation showed the strongest correlation with CAC-score. No correlation was found between CAC-score and endothelial dysfunction assessed by FMD. **Conclusions**: IMT, particularly when measured at the carotid bifurcation, was associated with CAC-score in women without diagnosed CAD, whereas no association was observed between FMD and CAC-score in this population.

## 1. Introduction

According to the ESC guidelines on cardiovascular disease prevention, during the assessment of risk for coronary artery disease (CAD), physicians may use additional diagnostic imaging methods, such as coronary artery calcium score (CAC-score), cardiac computed tomography angiography (CCTA), carotid ultrasound with measurement of the intima–media complex (IMT), ankle–brachial index (ABI), resting echocardiography, and assessment of arterial stiffness [1]. The American Heart Association (AHA) guidelines additionally mention exercise echocardiography, ischemia-induced artery dilatation (FMD) testing and myocardial perfusion imaging (MPI) [2]. The results of these tests may influence the final assessment of cardiovascular risk, either increasing or decreasing the patient’s individual risk [1,2]. According to the 2021 ESC Guidelines, CAC-score measurement may be considered when the calculated risk of cardiovascular disease reaches the threshold values for low and intermediate risk groups, allowing potential risk reclassification [1]. The AHA recommends CAC-score assessment in asymptomatic patients with intermediate cardiovascular risk (10-year risk of cardiovascular events). The test may also be considered in individuals at low-to-intermediate risk [2].

Measurement of the intima–media thickness (IMT) of the common carotid artery is used in the assessment of cardiovascular risk [3]. Guidelines recommend the IMT measurement when CT with CAC-score assessment is not possible, particularly in patients with intermediate cardiovascular risk. However, the lack of standardization of the examination methodology complicates the interpretation and comparison of studies using IMT for cardiovascular risk assessment [1]. Numerous studies indicate the potential usefulness of IMT in patients with borderline cardiovascular risk for reclassification [4,5]. The AHA guidelines recommend IMT measurement in asymptomatic patients with intermediate cardiovascular risk, emphasizing that it must be performed by an experienced ultrasonographer to ensure high-quality results [2].

However, despite existing recommendations, there is still no clear consensus regarding the optimal site of IMT measurement for the assessment of subclinical atherosclerosis. Different measurement locations may reflect different stages or characteristics of atherosclerotic changes, which justifies further investigation.

FMD is a recognized prognostic indicator of cardiovascular events [6,7,8,9,10,11]. Impaired endothelial function plays a significant role in the pathogenesis of CAD. It has also been shown that factors improving endothelial function may ultimately reduce cardiovascular risk [12,13,14]. Only a few studies suggest a correlation between FMD and CAC-score, and these are based on selectively chosen patient groups and may not be representative [15,16]. They are not very representative and have a selectively chosen group of patients [17,18]. The ESC guidelines do not mention FMD testing, while the AHA guidelines do not recommend its use for cardiovascular risk assessment in asymptomatic patients [2]. However, due to the limited and inconsistent data regarding the relationship between FMD and CAC-score, further investigation of this parameter may provide additional insight into its potential role in the assessment of subclinical atherosclerosis.

This study aimed to assess the correlation between the advancement of atherosclerotic lesions described by the CAC-score and the degree of IMT thickening of the common carotid artery in women. Moreover, we aimed to evaluate the relationship between the severity of coronary atherosclerosis based on CAC-score and endothelial dysfunction assessed by the FMD test. Additionally, we sought to determine the carotid artery site at which IMT measurement correlates most strongly with the advancement of atherosclerotic lesions described by the CAC-score in the study population.

## 2. Methods

### 2.1. The Study Population

The study population consisted of women who were routinely referred to the Department of Non-Invasive Diagnostic Imaging in Katowice for CAC-score measurement using CCTA. Ultimately, 124 women were included in the study. After enrollment, all participants were divided into three groups based on the CAC-score: study group A (100–400 AU), study group B (>400 AU), and control group (0 AU). Each participant had their medical history assessed to obtain information regarding age, weight, height, smoking, hypertension, diabetes, and lipid disorders. Then, ultrasound examinations were performed to measure IMT and FMD.

The protocol for this study was approved by the Bioethics Committee of the Medical University of Silesia in Katowice (approval no. PCN/CBN/0052/KB1/19/22). Inclusion and exclusion criteria are presented in Table 1.

### 2.2. The Coronary Artery Calcium Score (CAC-Score)

The patients underwent CCTA without the administration of a contrast agent to the vessels. The obtained image was divided into individual pixels. The threshold value for a single pixel was 130 HU. To reduce noise and errors, pixels must have an area of at least 1 mm^2^ to be classified as significant. The CT scan displayed all pixels that had a value of at least 130 HU and a size of 1 mm^2^. Each area with a radiological density of 130–199 HU received 1 point, those with 200–299 HU received 2 points, and those with 300–399 HU received 3 points. An area that reached a value ≥ 400 HU received 4 points. The above method was based on the analysis of an image composed of pixels and the summation of scores from all areas meeting the threshold values. All coronary arteries were assessed sequentially, and the obtained scores were summed into a single final score, reported in AU [19,20].

### 2.3. The Measurement of Carotid Intima–Media Complex (IMT)

The measurement of the carotid intima–media complex (IMT) refers to the assessment of its thickness in carotid arteries during ultrasound examination. In this study, measurements were taken within the distal carotid artery wall at two locations: at the bifurcation of the common carotid artery into the internal and external carotid arteries and 20 mm proximal to the bifurcation. The measurement line was perpendicular to the vessel wall. The carotid arteries were imaged during the end-diastolic phase, in both the long and short axes. At least two continuous image sequences (cineloops) and at least two single images (still images) of the measurement areas were recorded. Then, the average of the obtained IMT measurements was calculated. All measurements were performed on the same device by the same ultrasonographer [2,21].

### 2.4. The Ischemia-Induced Artery Dilatation Test (FMD)

The FMD test is an ultrasound examination that assesses changes in brachial artery dilatation in response to ischemia. Initially, blood pressure was measured by a sphygmomanometer on the selected upper limb. Then, the brachial artery was visualized in a longitudinal section with the help of ultrasound, on the same arm on which blood pressure was measured. The measurement of the arterial diameter was done at rest, during the end-diastolic phase. At least two continuous image sequences (cineloops) and two single images (still images) were recorded, and then the average of all measurements was calculated. In the second stage, the sphygmomanometer cuff was placed on the distal part of the chosen arm. Next, the cuff was inflated with air for 5 min to a value 50 mmHg higher than the patient’s systolic blood pressure. This procedure caused compression and transient ischemia and hypoxia of the distal part of the limb. After 60 s following the restoration of normal flow in the brachial artery (after a total of 6 min from the initial inflation of the sphygmomanometer cuff), the vessel diameter was measured again by recording at least two continuous image sequences (cineloops) as well as two single images (still images), and then the average of all measurements was calculated. FMD was determined by the following formula: FMD = (A − B)/B × 100% (A—vessel diameter 60 s after ischemia, B—vessel diameter before ischemia). In a healthy person with normal endothelial function, the examination should demonstrate ischemia-induced arterial dilatation by 7–10% compared to baseline values. Lesser or no dilatation may indicate impaired endothelial function [7,10,11].

### 2.5. Statistical Analysis

Statistical analysis was performed in RStudio (version 2024.12.1) using the R programming language. Descriptive analysis was presented in tables. Normality of variable distribution was assessed using the Shapiro–Wilk test, histograms, and Q–Q plots generated in RStudio. Correlation was assessed using the Spearman rank correlation coefficient. Principal component analysis (PCA) was used to isolate two factors, which were included in further analyses. The first dimension (PC1) consisted of IMT measurements at the bifurcation (IMT-L-bifurcation and IMT-P-bifurcation). The second dimension (PC2) consisted of IMT measurements 20 mm from the carotid bulb (IMT-L-20 mm and IMT-P-20 mm). The extracted factors accounted cumulatively for 85.9% of the total variance in the data. Relationships between variables were assessed using chi-square tests for qualitative variables, analysis of variance (ANOVA) for quantitative variables with a near-normal distribution, and the Kruskal–Wallis test for quantitative variables with a non-normal distribution (with a significance threshold of *p* < 0.05). Post hoc multiple comparisons were performed for significant relationships. Logistic regression models were constructed to assess the influence of variables on the assignment of patients to groups A, B, and the control group.

## 3. Results

### 3.1. Study Group Characteristic

Descriptive statistics for the studied variables, including cardiovascular risk factors such as BMI, smoking status, hypertension, diabetes, and lipid disorders, are presented in Table 2.

### 3.2. Main Findings

It was observed that the IMT values measured at the bifurcations and the PC1 factor describing them were significantly higher in groups A and B than in the control group, and significantly lower in group A than in group B. The IMT-P-20 mm and IMT-L-20 mm values, the PC2 factor describing them, and age were significantly higher in groups A and B than in the control group, but did not differ significantly between group A and group B. Statistical analysis did not detect significant differences between groups A, B, and the control group in the FMD parameter (*p* = 0.253). The above results are presented in Table 3.

Due to better model fit, the units of the four variables related to IMT measurement (IMT-L-bifurcation, IMT-P-bifurcation, IMT-L-20 mm, and IMT-P-20 mm) were multiplied by 100 (from cm to 0.1 mm). Univariate logistic regression analysis comparing group A with the control group showed that significantly higher values of IMT-L-bifurcation, IMT-P-bifurcation, PC1, and PC2 were observed in group A compared to the control group (Table 4). Based on these variables, a multivariate model was created. In the univariate model, a stronger association was observed between parameters describing IMT at the bifurcation than between those measured 20 mm from the bifurcation.

In univariate models describing the influence of variables on assignment to group B relative to the control group, a significant impact was observed for IMT-P and IMT-L values in relation to bifurcation, IMT-P-20 mm, age, PC1, and PC2 (Table 4). This demonstrates the potential for differentiation between the control group and group B, excluding group A.

In the univariate models describing the influence of variables on the assignment to group B in relation to group A, a significant impact of IMT-P and IMT-L values at the bifurcation and PC1 was observed (Table 4).

The multivariate model showed a significant influence of age, PC1, and PC2 on assignment to group A compared to the control group (Table 5). This allows us to conclude that, regardless of the age difference between the groups, described in Table 3, IMT parameters presented in the model as principal components have an impact on group membership. Principal component values (PC1 and PC2) were used in the model instead of raw IMT values due to strong correlations between individual IMT measurements (Table 6), which may lead to multicollinearity, potentially distorting the results.

In the multivariate model, a significant impact of age, PC1, and PC2 on assignment to group B was observed compared to the control group (Table 5). As in the previous case, this allows us to conclude that, regardless of the age difference between the groups, described in Table 3, IMT parameters presented in the model as principal components have an influence on group membership. In this model, principal component values were also used instead of raw IMT values due to the strong correlations between IMT measurements (Table 6), which may lead to multicollinearity, potentially confounding the results.

It was observed that patients in the control, A, and B groups differed significantly in terms of IMT parameters, with the values increasing from the control group to group B. These relationships were confirmed in logistic regression models, so in the next part of the analysis, an attempt was made to determine cutoff points for differentiating group affiliations based on the studied parameters. ROC analysis showed that measurements at the carotid bifurcation had better discriminatory ability than measurements obtained 20 mm from the carotid bulb. The best parameters were obtained at the right bifurcation (IMT-P-bifurcation), with a sensitivity of 83% and a specificity of 70% at a cutoff point of 0.07 cm. The results of the ROC analysis are presented in Table 7.

When differentiating between groups A and B for IMT-P-20 mm and IMT-L-bifurcation values, more than one potential cutoff point was obtained for the same AUC. Again, measurements at the common carotid bifurcation had a better ability to discriminate patients between groups than measurements at 20 mm from the carotid bulb (Table 8).

## 4. Discussion

This study demonstrated a significant association between ultrasound-derived IMT measurements in the carotid artery and CAC-score. Higher IMT values were observed in patients with higher CAC-scores, particularly for measurements obtained at the carotid bifurcation. These findings suggest that greater IMT thickening is associated with more advanced atherosclerosis in the studied population.

The scientific literature has repeatedly demonstrated a correlation between IMT measured by ultrasonography and CAC-score [22,23,24,25,26,27,28,29]. Other studies have also shown a correlation between IMT thickening and the occurrence of cardiovascular disease [30,31]. In a study by Barrett-Connor et al. [32], asymptomatic adults were examined, and a significant correlation was found between IMT and CAC-score. The highest specificity was observed in patients with a CAC-score < 400 AU. In a study by Davis et al. [33], a significant correlation was found between IMT and CAC-score among both young men (*p* < 0.025) and young women (*p* < 0.005) aged under 42 years. In a study by Newman et al. [34], a significant correlation between IMT and CAC-score was demonstrated among older adults without diagnosed coronary artery disease (mean age 79.9 ± 4, age range 70–97 years). Roy et al. [22] reported that most asymptomatic middle-aged women (50–69 years) without diagnosed coronary artery disease, but with at least one cardiovascular risk factor, presented with atherosclerotic plaques or increased IMT. Furthermore, all women with a CAC-score > 100 AU had IMT thickening of at least 0.15 cm on ultrasound examination. However, the literature also includes studies that do not confirm a correlation between CAC-score and IMT [35,36], as well as studies showing that CAC-score correlates more strongly with the presence of CAD than IMT [36,37,38]. Moreover, most available studies involve heterogeneous populations, and data focusing exclusively on women remain limited, as does evidence comparing different IMT measurement sites. Therefore, in the present study, we focused on a population of women and compared IMT measurements obtained at different carotid artery locations. Our results showed that IMT measured at the carotid bifurcation had a stronger association with CAC-score than those obtained 20 mm proximally. Furthermore, a potential cutoff point (0.07 cm) was identified. However, this finding should be interpreted with caution due to the limited sample size and lack of external validation. What is more, ROC analysis demonstrated that IMT measurement at the carotid artery bifurcation showed better discriminatory ability than measurements obtained at a distance of 20 mm from the carotid artery bifurcation for assigning patients to the appropriate groups: control, study group A, and study group B. This suggests that IMT measurements at the bifurcation are more precise in identifying significant changes that may correlate with a higher CAC-score. Furthermore, the above analysis yielded a cutoff point of 0.07 cm for abnormal IMT measurements at the bifurcation, which may be associated with a higher CAC-score. Measurements at the bifurcation in the right carotid artery were more sensitive than those in the left carotid artery. In the study by Takashi et al. [23], the authors found that IMT thickening of at least 0.07 cm correlates with CAD. In the study by Araki et al. [39], the authors demonstrated a correlation between automated measurement of coronary calcium volume in coronary vessels and automated measurement of IMT; measurements in the right carotid artery correlated more strongly than those in the left carotid artery. In the study by Cohen et al. [40], the authors showed that mean IMT values > 0.075 cm (three measurement sites—ICA, carotid bulb, and CCA) are associated with a CAC-score > 0 AU, regardless of patient age or gender. The above-mentioned studies and our analysis suggest that IMT measurement at the carotid bifurcation is associated with CAC-score in patients without previously diagnosed coronary artery disease.

Some cutoff points may be identified, but further research is needed to accurately establish appropriate guidelines. No association was observed between FMD and CAC-score in the present study. This finding may be explained by the fact that FMD reflects endothelial function, which represents an earlier and more functional stage of atherosclerosis, whereas CAC-score reflects more advanced structural changes. Additionally, FMD measurements are highly dependent on methodological factors, including patient preparation, environmental conditions, and operator experience, which may affect the sensitivity and reproducibility of the results.

## 5. Study Limitations

This study has several limitations that should be considered. First, the study population consisted exclusively of women, which may limit the generalizability of the results to other populations. Second, although IMT measurement is a standardized method, differences in measurement protocols and locations may influence the results and their interpretation.

Additionally, the study was conducted in a relatively small group of patients from a single center and had a cross-sectional design, which may affect the external validity of the findings and limit the ability to draw causal inferences. Moreover, the inclusion of patients referred for CAC assessment may introduce selection bias and limit the applicability of the findings to the general population. Furthermore, although multivariate analyses were performed, adjustment for all potential confounding factors was limited, and residual confounding by classical cardiovascular risk factors, particularly age, cannot be excluded.

Finally, the FMD test, although widely used in research settings, is characterized by high variability and operator dependence. In addition, several pre-analytical and methodological factors, such as patient preparation, dietary intake, medication use, and time of measurement, may influence the results, which could have affected the sensitivity of this parameter in the present study.

## 6. Conclusions

This study demonstrated a significant positive association between CAC-score and the degree of IMT complex thickening of the common carotid artery in women. Moreover, IMT measured at the carotid bifurcation showed the strongest association with CAC-score in this population. However, no association was found between CAC-score and endothelial dysfunction assessed by the FMD test in women.

Due to the cross-sectional design and the single-center nature of the study, these findings should be interpreted with caution and require confirmation in larger, prospective studies.

## Figures and Tables

**Table 1:** Inclusion and exclusion criteria.

Inclusion Criteria	Exclusion Criteria
Female gender	No written informed consent of the patient for participation in the study
Patient age ≥ 35 years old	Coronary artery disease or myocardial infarction diagnosed by diagnostic imaging test
Performed computed tomography with the coronary artery calcium score (CAC-score)	History of myocardial infarction
	Lack of complete patient information and necessary data during ultrasound examination

**Table 2:** Descriptive statistics of qualitative variables of the study group.

Variable	Group A n (%)/Mean (SD)	Group B n (%)/Mean (SD)	Control n (%)/Mean (SD)	Total n (%)/Mean (SD)
n	40	40	44	124
CAC-score [AU]	215 (77.22)	1128.13 (591.64)	0 (0)	433.30 (593.74)
IMT-P-bifurcation [cm]	0.11 (0.08)	0.17 (0.11)	0.06 (0.02)	0.11 (0.09)
IMT-P-20 mm [cm]	0.11 (0.08)	0.12 (0.09)	0.09 (0.06)	0.11 (0.08)
IMT-L-bifurcation [cm]	0.12 (0.10)	0.17 (0.12)	0.06 (0.02)	0.11 (0.10)
IMT-L-20 mm [cm]	0.11 (0.08)	0.11 (0.07)	0.09 (0.09)	0.11 (0.08)
Hypertension	33 (26.6)	36 (29.03)	27 (21.77)	96 (77.42)
Diabetes type 2	12 (9.68)	19 (15.32)	8 (6.45)	39 (31.45)
Smoking	8 (6.45)	13 (10.48)	9 (7.26)	30 (24.19)
Lipid disorders	28 (22.58)	33 (26.61)	25 (20.16)	86 (69.35)
Age [years]	69.9 (8.46)	70.08 (6.47)	49 (0)	66.21 (9.92)
BMI [weight/height^2^]	28.33 (5.67)	29.27 (6.18)	27.11 (5.66)	28.20 (5.86)

BMI—Body Mass Index; CAC-score—Coronary Artery Calcium score; IMT-P-bifurcation—thickness of the intima–media complex at the bifurcation of the right carotid artery; IMT-P-20 mm—thickness of the intima–media complex at a distance of 20 mm from the bifurcation of the right carotid artery; IMT-L-bifurcation—thickness of the intima–media complex at the bifurcation of the left carotid artery; IMT-L-20 mm—thickness of the intima–media complex at a distance of 20 mm from the bifurcation of the left carotid artery.

**Table 3:** Comparison of quantitative variables between groups (analysis of variance and Kruskal–Wallis tests *) with post hoc analysis of multiple comparisons.

Variable	Group	n	min	max	Median	Q1	Q3	*p*	Post Hoc
IMT-P-bifurcation [cm] *	A	40	0.05	0.38	0.08	0.07	0.12	<0.001	a, b, c
	B	40	0.05	0.47	0.15	0.074	0.229		
	control	44	0.03	0.09	0.055	0.045	0.07		
IMT-P-20 mm [cm] *	A	40	0.04	0.5	0.085	0.06	0.14	0.005	a, b
	B	40	0.04	0.38	0.08	0.07	0.124		
	control	44	0.04	0.38	0.065	0.059	0.085		
IMT-L-bifurcation [cm] *	A	40	0.045	0.48	0.08	0.069	0.1	<0.001	a, b, c
	B	40	0.04	0.45	0.135	0.08	0.23		
	control	44	0.04	0.12	0.06	0.05	0.07		
IMT-L-20 mm [cm] *	A	40	0.04	0.415	0.08	0.07	0.136	0.033	a, b
	B	40	0.03	0.32	0.09	0.074	0.149		
	control	44	0.04	0.6	0.07	0.06	0.086		
FMD [%]	A	40	0	11.429	5.263	2.819	7.143	0.152	
	B	40	0	14.286	3.935	2.615	6.505		
	control	44	1.37	14.286	5.971	3.008	8.194		
Age [years]	A	40	52	89	72	65	75	<0.001	a, b
	B	40	57	83	70	68	74.25		
	control	44	36	80	59	53.75	68		
PC1 *	A	40	−0.163	0.4	−0.04	−0.066	0.009	<0.001	a, b, c
	B	40	−0.126	0.484	0.053	−0.055	0.152		
	control	44	−0.122	−0.015	−0.071	−0.08	−0.054		
PC2 *	A	40	−0.107	0.421	−0.014	−0.057	0.039	<0.001	a, b
	B	40	−0.094	0.279	0.015	−0.037	0.076		
	control	44	−0.112	0.306	−0.064	−0.08	−0.033		

a—Significant difference between group A and the control group; b—significant difference between group B and the control group; c—significant difference between group A and B. PC1—Principal component 1; PC2—principal component 2; FMD—Flow-Mediated Dilation; IMT-L-bifurcation—thickness of the intima–media complex at the bifurcation of the left carotid artery; IMT-L-20 mm—thickness of the intima–media complex at a distance of 20 mm from the bifurcation of the left carotid artery; IMT-P-bifurcation—thickness of the intima–media complex at the bifurcation of the right carotid artery; IMT-P-20 mm—thickness of the intima–media complex at a distance of 20 mm from the bifurcation of the right carotid artery.

**Table 4:** Univariate models analyzing the influence of variables on assignment to respective groups.

Variable	Odds Ratio	Standard Error	*p*
Univariate models analyzing the influence of variables on assignment to group A in relation to the control group.			
IMT-P-bifurcation [0.1 mm]	2.344	0.209	<0.001
IMT-P-20 mm [0.1 mm]	1.063	0.039	0.122
IMT-L-bifurcation [0.1 mm]	1.586	0.145	0.001
IMT-L-20 mm [0.1 mm]	1.031	0.028	0.284
Age [years]	1.129	0.03	<0.001
PC1	1.275	0.084	0.004
PC2	1.065	0.029	0.032
Univariate models analyzing the influence of variables on assignment to group B in relation to the control group.			
IMT-P-bifurcation [0.1 mm]	1.798	0.172	0.001
IMT-P-20 mm [0.1 mm]	1.079	0.037	0.042
IMT-L-bifurcation [0.1 mm]	1.669	0.15	0.001
IMT-L-20 mm [0.1 mm]	1.034	0.031	0.29
Age [years]	1.16	0.035	<0.001
PC1	1.224	0.055	<0.001
PC2	1.116	0.034	0.001
Univariate models analyzing the influence of variables on assignment to group B in relation to group A.			
IMT-P-bifurcation [0.1 mm]	1.064	0.027	0.023
IMT-P-20 mm [0.1 mm]	1.014	0.027	0.606
IMT-L-bifurcation [0.1 mm]	1.05	0.023	0.036
IMT-L-20 mm [0.1 mm]	0.997	0.031	0.914
Age [years]	1.003	0.03	0.916
PC1	1.038	0.018	0.033
PC2	1.025	0.024	0.31

IMT-L-bifurcation—Thickness of the intima–media complex at the bifurcation of the left carotid artery; IMT-L-20 mm—thickness of the intima–media complex at a distance of 20 mm from the bifurcation of the left carotid artery; IMT-P-bifurcation—thickness of the intima–media complex at the bifurcation of the right carotid artery; IMT-P-20 mm—thickness of the intima–media complex at a distance of 20 mm from the bifurcation of the right carotid artery; IMT—intima–media thickness; PC1—principal component 1; PC2—principal component 2.

**Table 5:** Multivariate models analyzing the influence of variables on assignment to respective groups.

Variable	Odds Ratio	Standard Error	*p*	95% CI	
A multivariate model analyzing the influence of variables on assignment to group A in relation to the control group.					
Age [years]	1.109	0.034	0.002	1.042	1.194
PC1	1.871	0.206	0.002	1.336	3.018
PC2	1.172	0.055	0.004	1.065	1.325
A multivariate model analyzing the influence of variables on assignment to group B in relation to the control group.					
Age [years]	1.190	0.066	0.008	1.062	1.389
PC1	1.705	0.203	0.009	1.269	2.798
PC2	1.242	0.071	0.002	1.105	1.472

PC1—Principal component 1; PC2—principal component 2.

**Table 6:** Table showing correlations between IMT measurements and CAC-score (Spearman correlation coefficient).

IMT-P								
Correlations	IMT-P- bifurcation [cm]		IMT-P- bifurcation [mm]		IMT-P-20 mm [cm]		IMT-P-20 mm [mm]	
	*p*	R	*p*	R	*p*	R	*p*	R
CAC-score [AU]	0.006	0.305	0.006	0.305	0.848	0.022	0.848	0.022
IMT-L								
Correlations	IMT-L- bifurcation [cm]		IMT-L- bifurcation [mm]		IMT-L-20 mm [cm]		IMT-L-20 mm [mm]	
	*p*	R	*p*	R	*p*	R	*p*	R
CAC-score [AU]	0.002	0.340	0.002	0.340	0.448	0.086	0.448	0.086

CAC-score—Coronary Artery Calcification score. IMT-P-bifurcation—thickness of the intima–media complex at the bifurcation of the right carotid artery. IMT-P-20 mm—thickness of the intima–media complex at a distance of 20 mm from the bifurcation of the right carotid artery. IMT-L-bifurcation—thickness of the intima–media complex at the bifurcation of the left carotid artery. IMT-L-20 mm—thickness of the intima–media complex at a distance of 20 mm from the bifurcation of the left carotid artery.

**Table 7:** ROC analysis with cutoff points between group A and the control group.

Variable	Cutoff Point	Specificity	Sensitivity	npv	ppv	AUC
IMT-P-bifurcation [cm]	0.07	0.70	0.83	0.82	0.72	0.86
IMT-P-20 mm [cm]	0.08	0.66	0.65	0.67	0.63	0.65
IMT-L-bifurcation [cm]	0.07	0.80	0.63	0.70	0.74	0.78
IMT-L-20 mm [cm]	0.105	0.86	0.4	0.61	0.73	0.61

IMT-L-bifurcation—Thickness of the intima–media complex at the bifurcation of the left carotid artery; IMT-L-20 mm—thickness of the intima–media complex at a distance of 20 mm from the bifurcation of the left carotid artery; IMT-P-bifurcation—thickness of the intima–media complex at the bifurcation of the right carotid artery; IMT-P-20 mm—thickness of the intima–media complex at a distance of 20 mm from the bifurcation of the right carotid artery; npv—negative predictive value; ppv—positive predictive value; AUC—area under the curve.

**Table 8:** ROC analysis to differentiate between group A and B.

Variable	Cutoff Point	Specificity	Sensitivity	npv	ppv	AUC
IMT-P-bifurcation [cm]	0.14	0.80	0.55	0.64	0.73	0.64
IMT-P-20 mm [cm]	0.09	0.40	0.68	0.55	0.53	0.48
	0.08	0.53	0.55	0.54	0.54	
IMT-L-bifurcation [cm]	0.11	0.78	0.58	0.65	0.72	0.67
	0.12	0.80	0.55	0.64	0.73	
	0.15	0.85	0.50	0.63	0.77	
IMT-L-20 mm [cm]	0.07	0.43	0.75	0.63	0.57	0.54

IMT-L-bifurcation—Thickness of the intima–media complex at the bifurcation of the left carotid artery; IMT-L-20 mm—thickness of the intima–media complex at a distance of 20 mm from the bifurcation of the left carotid artery; IMT-P-bifurcation—thickness of the intima–media complex at the bifurcation of the right carotid artery; IMT-P-20 mm—thickness of the intima–media complex at a distance of 20 mm from the bifurcation of the right carotid artery.

## Data Availability

The data presented in this study are available on request from the corresponding author.

## Abbreviations

ABI	Ankle–brachial index
AHA	American Heart Association
AU	Agatston unit
CAC-score	Coronary Artery Calcification score
CAD	Coronary artery disease
CCA	Common carotid artery
CCTA	Cardiac computed tomography angiography
ESC	European Society of Cardiology
FMD	Flow-mediated dilation
HU	Hounsfield unit
ICA	Internal carotid artery
IMT-L-bifurcation	Thickness of the intima–media complex at the bifurcation of the left carotid artery
IMT-L-20 mm	Thickness of the intima–media complex at a distance of 20 mm from the bifurcation of the left carotid artery
IMT-P-bifurcation	Thickness of the intima–media complex at the bifurcation of the right carotid artery
IMT-P-20 mm	Thickness of the intima–media complex at a distance of 20 mm from the bifurcation of the right carotid artery
IMT	Intima–media thickness
MPI	Myocardial perfusion imaging
PC 1	Principal component 1
PC 2	Principal component 2
PCA	Principal Component Analysis
qq diagram	Quantile–quantile diagram
